# Direct Observation of Electric‐Field‐Driven Phase Transitions Associated with Energy Storage in Antiferroelectric Films

**DOI:** 10.1002/advs.202517897

**Published:** 2025-12-21

**Authors:** Yan‐Peng Feng, Mei‐Xiong Zhu, Ru‐Jian Jiang, Yu‐Jia Wang, Yun‐Long Tang, Yin‐Lian Zhu, Xiu‐Liang Ma

**Affiliations:** ^1^ Bay Area Center for Electron Microscopy Songshan Lake Materials Laboratory Dongguan Guangdong China; ^2^ Quantum Science Center of Guangdong–HongKong–Macao Greater Bay Area Shenzhen China; ^3^ Shenyang National Laboratory for Materials Science Institute of Metal Research Chinese Academy of Sciences Shenyang China; ^4^ School of Materials Science and Engineering University of Science and Technology of China Shenyang China; ^5^ School of Materials Science and Engineering Hunan University of Science and Technology Xiangtan China; ^6^ Institute of Physics Chinese Academy of Sciences Beijing China; ^7^ State Key Lab of Advanced Processing and Recycling on Non‐ferrous Metals Lanzhou University of Technology Lanzhou China

**Keywords:** antiferroelectric films, antiferroelectric‐to‐ferroelectric phase transitions, external electric field, in situ transmission electron microscopy

## Abstract

Antiferroelectric materials are promising candidates for high‐energy‐density capacitors due to their reversible electric‐field‐induced phase transitions. However, the atomic‐scale mechanism underlying the electric‐field‐driven antiferroelectric‐to‐ferroelectric (AFE‐to‐FE) transition, particularly in relation to high‐performance energy storage, remains elusive. Here, we employ in situ aberration‐corrected scanning transmission electron microscopy (STEM) to directly visualize the electric‐field‐driven AFE‐to‐FE transition in epitaxial PbZrO_3_ (PZO) thin films. We reveal a sequential electric‐field‐driven transition pathway involving an intermediate orthorhombic ferrielectric phase (FiE_O_) and a monoclinic ferroelectric phase (FE_M_), ultimately stabilizing into a rhombohedral ferroelectric structure (FE_R_). This transformation is accompanied by a continuous reduction in the polarization modulation period, indicating enhanced dipole–dipole interaction coupling, which may improve their energy storage performance. Our experimental findings are corroborated by machine learning molecular dynamics simulations, providing quantitative insights into the structural evolution. This work illustrated the relationship between microscopic phase evolution dynamics and macroscopic energy storage behavior, offering a powerful strategy for the design and optimization of next‐generation antiferroelectric energy storage materials.

## Graphical abstract

The electric‐field‐driven antiferroelectric‐to‐ferroelectric phase transition in epitaxial PbZrO_3_ films is directly visualized by in situ aberration‐corrected scanning transmission electron microscopy. A sequential transition pathway involving an orthorhombic ferrielectric phase and a monoclinic ferroelectric phase, ultimately stabilizing into a rhombohedral ferroelectric structure is revealed, which is accompanied by a reduction in polarization modulation period, indicating enhanced dipole–dipole interaction.

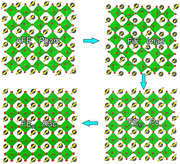

## 1 Introduction

Antiferroelectrics featuring antiparallel electric dipoles of equal magnitude possess zero net polarization in the absence of an electric field [1]. The critical feature of antiferroelectric materials is the macroscopic double hysteresis loop in the polarization‐electric field (P‐E) response. When an electric field is applied, the antiparallel dipoles can be reoriented in the same direction, which leads to antiferroelectric‐to‐ferroelectric (AFE‐to‐FE) phase transition [2, 3, 4]. This field‐induced phase transition enables significant charge storage, lattice expansion, and temperature cooling, and hence the antiferroelectric materials have wide applications in high‐density energy storage capacitors [5, 6, 7], electromechanical transducers [8, 9], and electrocaloric refrigeration [10, 11]. The AFE‐to‐FE phase transition process plays a critical role in the energy storage density (W) and efficiency (η), which are two significant factors in assessing the energy storage performance of antiferroelectric materials [12]. Therefore, a fundamental understanding of the electric‐field‐induced phase transition is essential for manipulating the energy storage performance of antiferroelectric materials.

PbZrO_3_ (PZO) is widely regarded as the prototypical antiferroelectric material since Kittel first proposed the theoretical concept of antiferroelectricity in 1951^1^. At the ground state, PZO generally displays an orthorhombic *Pbam* phase with the characteristic up‐up‐down‐down (↑↑↓↓) antipolar pattern of Pb cation displacements [13, 14, 15]. However, recent first‐principles calculations have challenged this consensus, suggesting that the true ground state of PZO may be ferrielectric rather than antiferroelectric [16]. Experimentally, a ferrielectric phase with an up‐up‐down (↑↑↓) dipole configuration has been observed in both single crystals [17] and thin films [18] of PZO, and this phase can be stabilized through strain‐mediated phase separation [19]. Although the AFE‐to‐FE phase transition has been extensively investigated by chemical doping [12, 20], film thickness [21], in situ synchrotron X‐ray diffraction [22], and electron beam irradiation [18, 23, 24], the directly electric‐field‐driven AFE‐to‐FE phase transition associated with energy storage behavior has remained poorly understood. In situ aberration‐corrected scanning transmission electron microscopy (STEM) under electrical bias has emerged as a powerful tool to probe topological phase transitions [25, 26], domain nucleation and switching behaviors [27, 28, 29] in ferroelectric thin films, thereby offering a promising avenue to explore the electric‐field‐driven AFE‐to‐FE transition at the atomic scale.

In this work, the electric‐field‐driven AFE‐to‐FE phase transition in epitaxial PZO films was investigated by in situ aberration‐corrected STEM. The PZO films, grown on a SrRuO_3_‐buffered KTaO_3_ (SRO/KTO) substrate, exhibit the presence of antiferroelectric phases at the initial state. By analyzing fast Fourier transform (FFT) patterns, Pb cation displacement vectors, and in‐plane lattice rotations, we elucidate the pathway of the electric‐field‐driven AFE‐to‐FE phase transition. Specifically, with increasing applied voltage, the antiferroelectric phase undergoes a transition successively through a ferrielectric state and a monoclinic ferroelectric phase, eventually transforming completely into a rhombohedral ferroelectric phase. This transition sequence is further corroborated by machine learning molecular dynamics simulations. Meanwhile, the phase transition is accompanied by a progressive reduction in the polarization modulation period. The electric‐field‐driven structural evolution observed here could be linked to the excellent energy storage performance of PZO films, providing crucial insights into the rational design and optimization of high‐density antiferroelectric energy storage materials.

## 2 Results and Discussion

**FIGURE 1:**
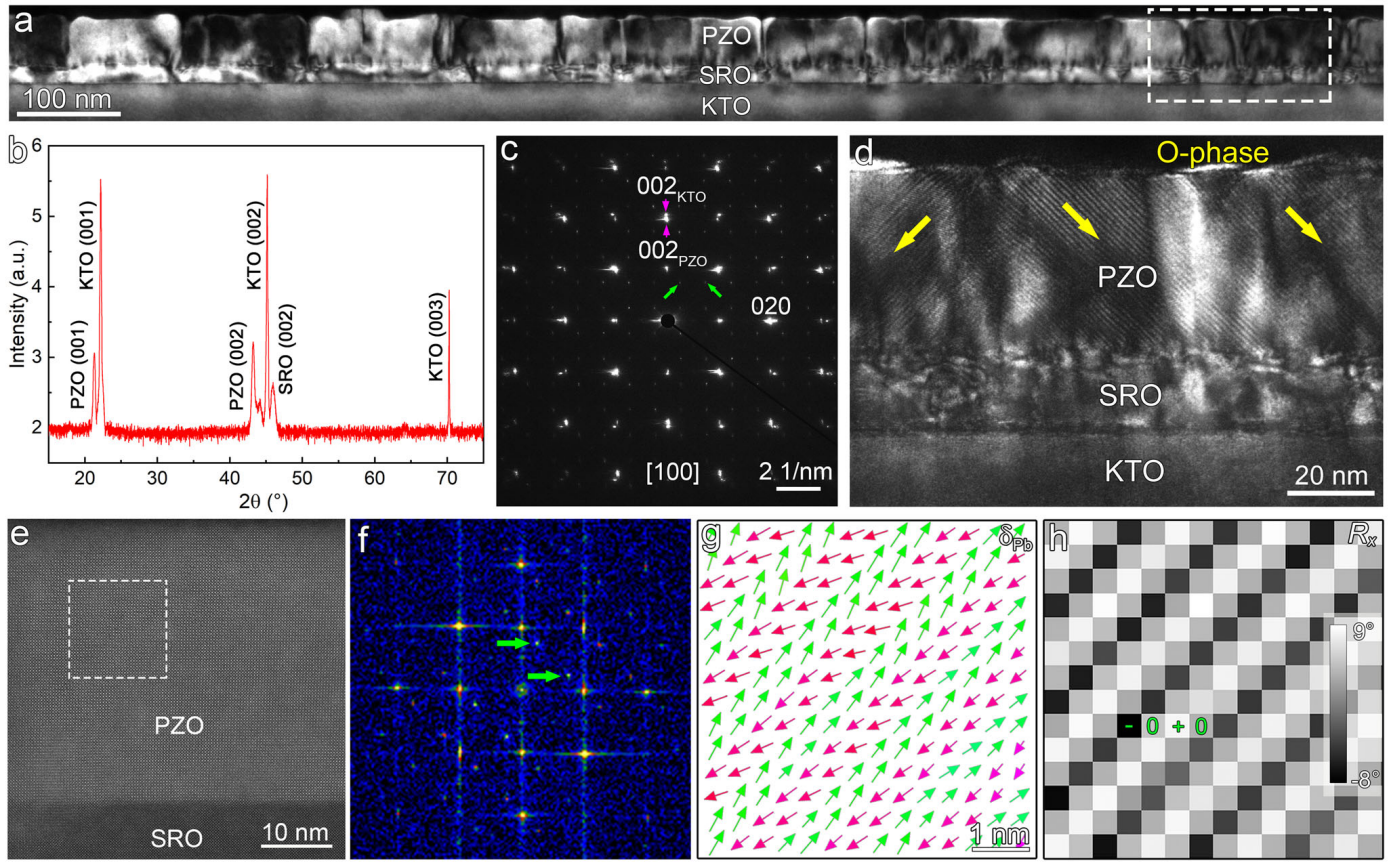
The microstructures and polarization characteristics of the PZO/SRO/KTO thin film in the initial state. (a) Cross‐sectional dark‐field TEM image of the PZO/SRO/KTO film. (b) XRD θ−2θ scan pattern of the PZO/SRO/KTO film. All crystallographic indices are referenced to the pseudocubic unit cell for simplicity. (c) SAED pattern acquired along the [100] zone axis, including both the KTO substrate and PZO/SRO film. The green arrows denote the 1/4{110} superlattice reflections. (d) Magnified dark‐field TEM image of the region marked by the dashed box in (a), revealing inclined stripe contrast along <110> directions (yellow arrows). (e) Atomic‐resolution HAADF‐STEM image of PZO layer. (f–h) The FFT pattern, Pb cation displacement (**δ_Pb_**) mapping, and in‐plane lattice rotation (*R_x_*) mapping corresponding to the region marked by dashed boxes in (e), respectively.

A 50 nm thick PZO thin film was epitaxially grown on SRO‐buffered [001]‐oriented KTO substrate using pulsed laser deposition (film deposition details are provided in Experimental Section). A low‐magnification cross‐sectional dark‐field TEM image (Figure 1a) reveals that the PZO layer exhibits a columnar growth morphology with high‐density misfit dislocations observed at the PZO/SRO interface, which might be ascribed to the large lattice mismatch between the PZO film and KTO substrate [13, 30]. The X‐ray diffraction (XRD) θ−2θ scan in the range of 15°–75° (Figure 1b) exhibits only the main diffraction peaks corresponding to the PZO and SRO layers, as well as the KTO substrate, which indicates that the films have a well crystallinity and no impure phases. In this work, all crystallographic indices are referenced to the pseudocubic unit cell for simplicity. The selected area electron diffraction (SAED) pattern (Figure 1c) shows an obvious splitting of the out‐of‐plane (002) diffraction spots. Besides, the 1/4{110} superlattice reflections (marked by green arrows) are clearly observed along both the [011] and [0$\bar{1}$1] directions corresponding to the anti‐parallel configurations of dipoles, indicating the antiferroelectric nature in the PZO films. A magnified view of the region highlighted by a dashed rectangle in Figure 1a is shown in Figure 1d, showing distinctive inclined stripes along the <110> directions (indicated by yellow arrows) within the PZO layer. These features could be indicative of an orthorhombic antiferroelectric phase (AFE_O_) as reported previously [18]. The atomic‐resolution HAADF‐STEM image (Figure 1e) shows high crystallization quality of the PZO film. The corresponding fast Fourier transform (FFT) pattern (Figure 1f) obtained from the region marked by a dashed rectangle in Figure 1e reveals additional 1/4{110} diffraction spots (highlighted by green arrows). To further characterize the region, the Pb cation displacement vector (**δ_Pb_**) and in‐plane lattice rotation (*R_x_*) mappings were shown in Figure 1g,h, respectively. The **δ_Pb_** mapping demonstrates a quadruple modulation period with ↑↑↓↓ polarization ordering, while the *R_x_* mapping exhibits an alternating (−, 0, +, 0) rotation pattern. The combined analysis of the FFT pattern, **δ_Pb_**, and *R_x_* mappings confirms the presence of the orthorhombic antiferroelectric phase in the PZO film.

To evaluate the phase transition behaviors, the in situ electric bias was conducted on cross‐sectional TEM samples to reveal the evolution of AFE_O_ phase in PZO films via a tungsten (W) tip of a double‐tilt in situ TEM holder. The low‐magnification high‐resolution HAADF‐STEM imaging under different electric voltages (0–12 V) were acquired along [100] directions using an aberration‐corrected scanning transmission electron microscope. A chronological series of high‐resolution HAADF‐STEM images (Figure S1) and corresponding FFT patterns (Figure S2) revealed a multi‐stage phase transition pathway. Initially, the exclusively additional 1/4{110} diffraction spots exist at 4 V (Figure S2b), indicating that the AFE_O_ phase persists up to 4 V. Upon reaching 5 V (Figure S2c), emergent 1/3{110} satellite spots (marked by red arrows) signal the onset of a first intermediate phase (IP_1_). At 7 V (Figure S2d), complete disappearance of 1/4{110} spots and emergence of 1/2{110} superlattice reflections (yellow arrows) indicate the formation of a second intermediate phase (IP_2_). When the voltage is continually increased, the 1/3{110} diffraction spots are progressively attenuated until their complete disappearance at 9 V (Figure S2f). Only the 1/2{110} superlattice reflections exist when the electric voltage is increased to 10 V. Finally, all superlattice features vanish at 12 V, which could enable a full transition to the ferroelectric phase. This sequential evolution (AFE_O_ → IP_1_ → IP_2_ → FE) demonstrates two distinct intermediate states during electric‐field‐driven AFE‐to‐FE phase transition, characterized by progressive superlattice diffraction spot transitions from 1/4{110}, to 1/3{110}, to 1/2{110}, and finally to fundamental perovskite patterns, which reflect symmetric modifications of PZO unit cells under electric field.

**FIGURE 2:**
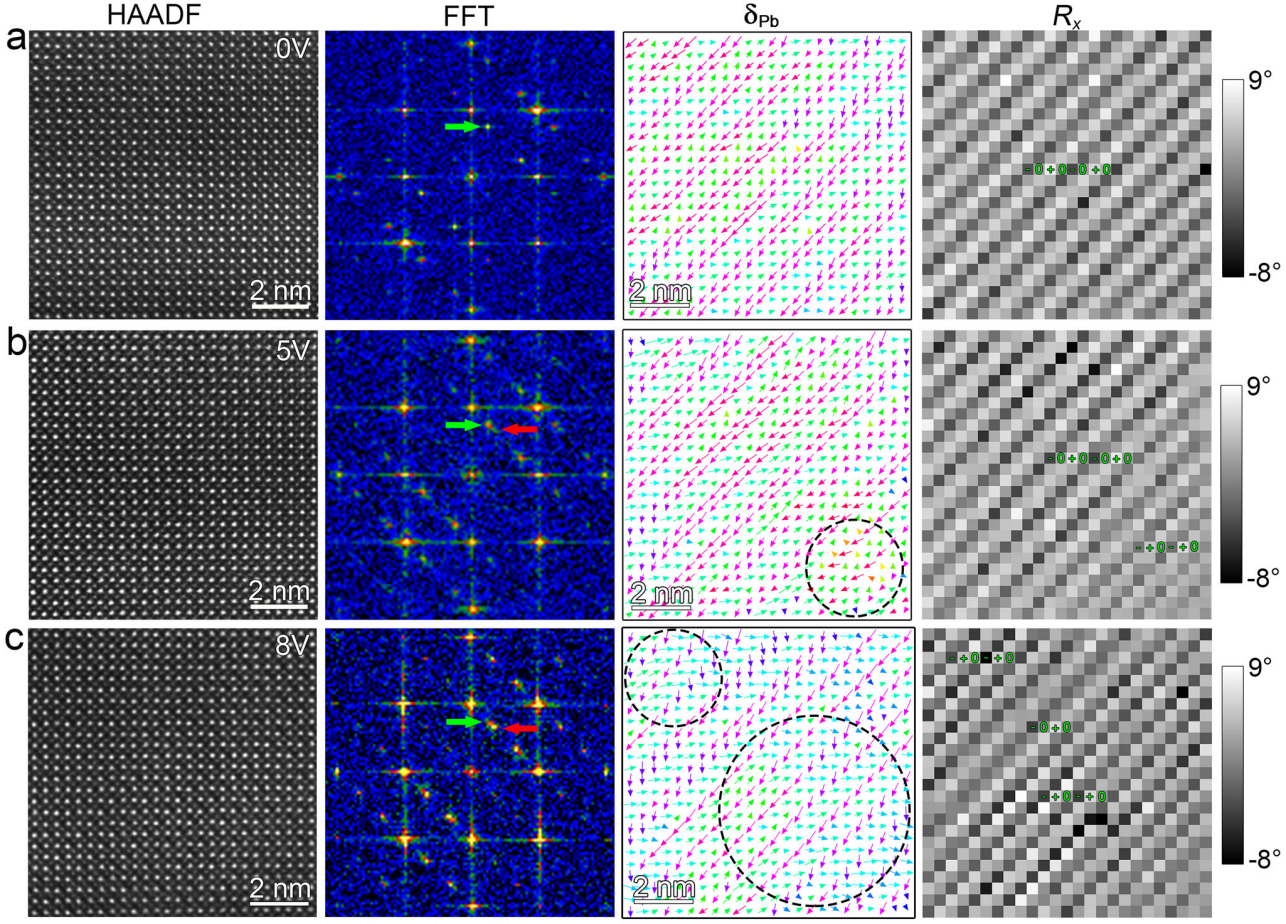
In situ atomic resolution HAADF‐STEM images to reveal the process of AFE_O_‐to‐FiE_O_ phase transition under an external electric field. (a) The atomic resolution HAADF‐STEM image and the corresponding FFT pattern, Pb‐displacement mapping (**δ_Pb_**), in‐plane lattice rotation mapping (*R_x_*) of PZO films at 0 V. (b) The atomic resolution HAADF‐STEM image and the corresponding FFT pattern, Pb‐displacement mapping (**δ_Pb_**), in‐plane lattice rotation mapping (*R_x_*) of PZO films at 5 V. The black circle denotes the AFE_O_‐to‐FiE_O_ phase transition starts to occur at this region. (c) The atomic resolution HAADF‐STEM image and the corresponding FFT pattern, Pb‐displacement mapping (**δ_Pb_**), in‐plane lattice rotation mapping (*R_x_*) of PZO films at 8 V. The black circles denote the PZO unit cells have transformed into FiE_O_ phase in these regions.

The atomic‐resolution HAADF‐STEM imaging under applied bias was performed to further identify the intermediate phase structures and elucidate the dynamics of electric‐field‐induced phase transitions in PZO films. Initially, an atomic‐resolution HAADF‐STEM image was acquired at 0 V, as shown in Figure S3a. The corresponding FFT pattern is displayed in the inset. A green arrow highlights the presence of an additional 1/4{110} reflection, which is a typical fingerprint of the antiferroelectric orthorhombic (AFE_O_) phase. Figure S3b shows the Pb‐displacement vector (**δ_Pb_**) mapping in the region outlined by the dashed red rectangle in Figure S3a. This mapping reveals that the region is predominantly occupied by the AFE_O_ phase, characterized by a quadruple‐modulated polarization configuration (↑↑↓↓). A magnified view of a typical region (white rectangle in Figure S3a) is shown in Figure 2a, alongside its corresponding FFT pattern, **δ_Pb,_** and *R_x_* mappings. The **δ_Pb_** mapping confirms the presence of the ↑↑↓↓ polarization configuration, while the *R_x_* mapping reveals alternating lattice rotation patterns of (−, 0, +, 0). Collectively, these results confirm that the PZO film in this region adopts the AFE_O_ phase at 0 V.

The applied electric field was increased to 5 V to capture the first stage phase transition. The atomic‐resolution HAADF‐STEM image at 5 V was shown in Figure S4a. The inset FFT pattern reveals the appearance of a 1/3{110} superlattice reflection (red arrows), coexisting with the previously observed 1/4{110} reflections. The **δ_Pb_** mapping for the region marked by the dashed red rectangle in Figure S4a is shown in Figure S4b. Here, the upper region maintains the AFE_O_ phase with a ↑↑↓↓ polarization pattern, whereas the lower‐right area (marked by dashed circles) shows a triple‐modulated ↑↑↓ configuration. Similarly, A magnified view of the region marked by the white rectangle in Figure S4a is presented in Figure 2b, along with its FFT pattern,** δ_Pb_**, and *R_x_* mappings. The **δ_Pb_** mapping reveals clear evidence of ↑↑↓ polarization modulation in the lower‐right region (black dashed circle). Furthermore, some polarization patterns (yellow arrows) have rotated toward the vertical direction. The *R_x_* mapping exhibits a triple modulation period with (−, +, 0). By the combined analysis of the FFT pattern, **δ_Pb_** and *R_x_* mappings, the intermediate phase (IP_1_) is identified as an orthorhombic ferrielectric (FiE_O_) phase with space group of *Ima*2, as reported in previous literature [16, 17, 18, 19]. This suggests that the AFE_O_ phase starts to transform into the FiE_O_ phase at 5 V.

Upon further increasing the electric field to 8 V, the AFE_O_‐to‐FiE_O_ phase transition continues. The atomic‐resolution HAADF‐STEM image at 8 V was shown in Figure S5a. The corresponding FFT pattern (the inset of Figure S5a) shows the coexistence of 1/4{110} and 1/3{110} superlattice reflections. Notably, the 1/3{110} reflections exhibit stronger intensity compared to those observed at 5 V, indicating an increased volume fraction of the FiE_O_ phase. Figure S5b shows the **δ_Pb_** mapping corresponding to the region labeled as a dashed red rectangle box in Figure S4a. It clearly reveals a widespread ↑↑↓ configuration across both the upper and lower regions, with only a small portion of PZO unit cells retaining the original ↑↑↓↓ AFE_O_ configuration. Similarly, the region marked by a white rectangle box in Figure S5a was magnified, and the corresponding FFT pattern, **δ_Pb_**, and *R_x_* mappings are shown in Figure 2c. Comparison of the FFT patterns across voltages reveals a progressive enhancement in the intensity of the 1/3{110} reflections, confirming the propagation of the FiE_O_ phase. The **δ_Pb_** and *R_x_* mappings further illustrate the spatial expansion of the FiE_O_ phase under increasing electric field. Therefore, the real‐time visualization and corresponding structural analysis demonstrate that the AFE_O_‐to‐FiE_O_ phase transition proceeds through nucleation and gradual expansion of the FiE_O_ phase under increasing electric field.

**FIGURE 3:**
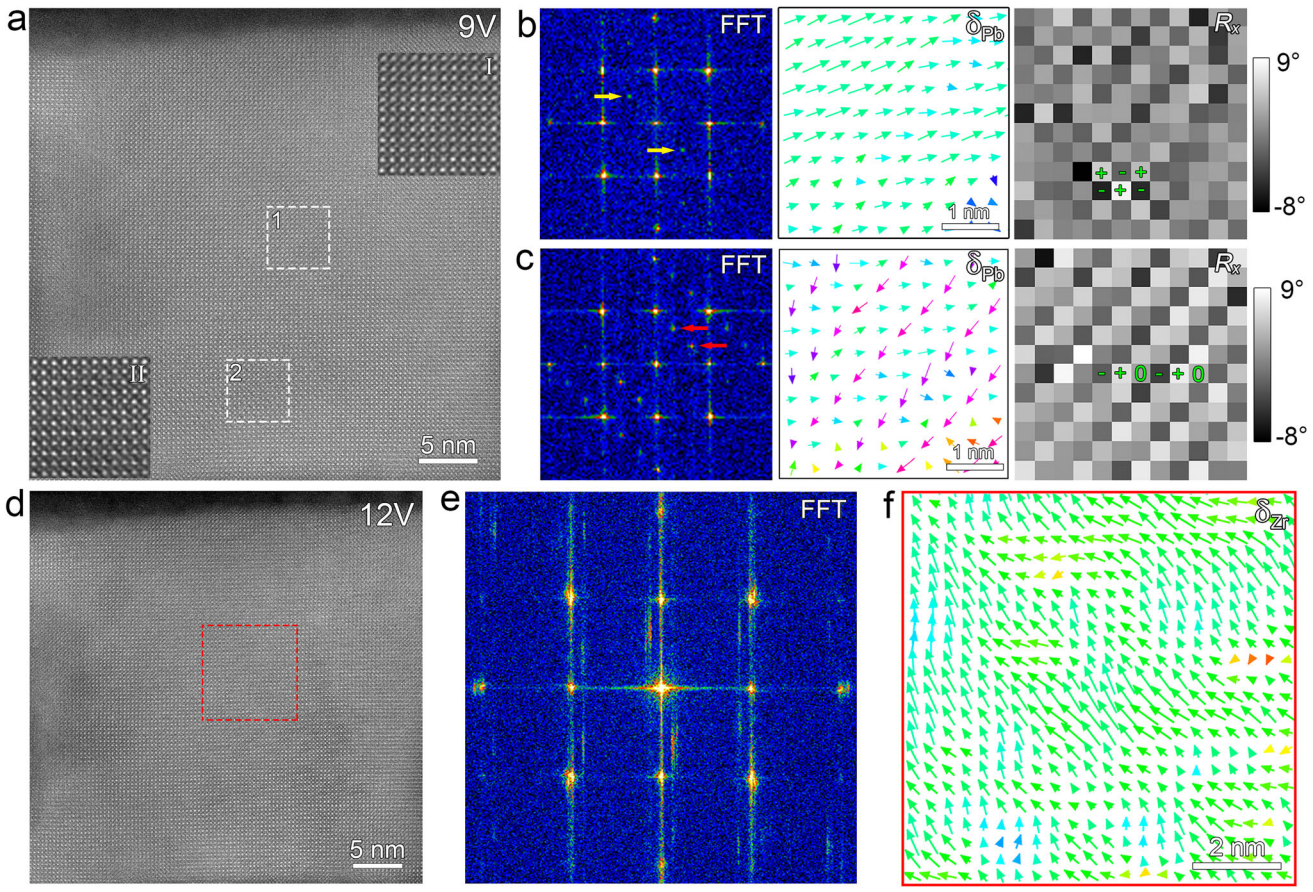
The FiE_O_‐FE_M_‐FE_R_ phase transition under further increasing the electric field. (a) The atomic‐resolved HAADF‐STEM image of PZO films at 9 V. The insets are two magnified HAADF‐STEM images corresponding to the regions labeled as dashed boxes numbered “1‐2”. (b) The FFT pattern, **δ_Pb_** and *R_x_* mappings corresponding to region 1. (c) The FFT pattern, **δ_Pb_** and *R_x_* mappings corresponding to region 2. (d) The atomic‐resolved HAADF‐STEM image of PZO films at 12 V. (e) The FFT pattern corresponding to the area of (d). (f) The **δ_Zr_** mapping corresponding to the region marked by the red dashed rectangle box in (d).

The applied voltage was further increased to observe the subsequent transformation of the FiE_O_ phase. The atomic‐resolution HAADF‐STEM image acquired at 9 V is presented in Figure 3a. Two typical regions (marked by the dashed white boxes “1‐2”) were selected, and their magnified images were displayed in the insets labeled as “I‐II” of Figure 3a, respectively. Figure 3b shows the FFT pattern, **δ_Pb_**, and *R_x_* mappings corresponding to region 1. The FFT pattern reveals emergent 1/2{110} superlattice reflections (marked by yellow arrows). The **δ_Pb_** mapping reveals a polarization ordering configuration deviating from <111> directions. The checkerboard‐like pattern in the *R_x_* mapping indicates a zigzag position of Pb cations. These structural features confirm that the IP_2_ is a monoclinic ferroelectric (FE_M_) phase with a space group of *Pc*, as previously reported in the literature [31]. Similarly, the FFT pattern, **δ_Pb_**, and *R_x_* mappings corresponding to region 2 are shown in Figure 3c. Region 2 retains 1/3{110} reflections (marked by red arrows), along with a typical triple‐modulated polarization and in‐plane lattice rotation, indicating that it remains in the FiE_O_ phase. These results suggest that the upper PZO has transformed into the FE_M_ phase, and the lower PZO still remains the FiE_O_ phase at 9 V, signifying an ongoing FiE_O_‐to‐FE_M_ phase transition. Upon increasing the voltage to 12 V, a complete phase transformation was observed. The atomic‐resolution HAADF‐STEM image at this voltage is shown in Figure 3d. The corresponding FFT pattern (Figure 3e) exhibits the absence of additional superlattice reflections, indicating that the PZO film has fully transitioned into a rhombohedral ferroelectric (FE_R_) phase. This phase is further supported by the Zr‐displacement (**δ_Zr_**) mapping in Figure 3f, which reveals polarization vectors aligning along the <111> directions, characteristic of the FE_R_ phase. However, high voltage can introduce more scanning noise, leading to dynamic fluctuations in the polarization vectors (Figure 3f), which is further evidenced by the appearance of streaking artifacts in the FFT pattern (Figure 3e).

**FIGURE 4:**
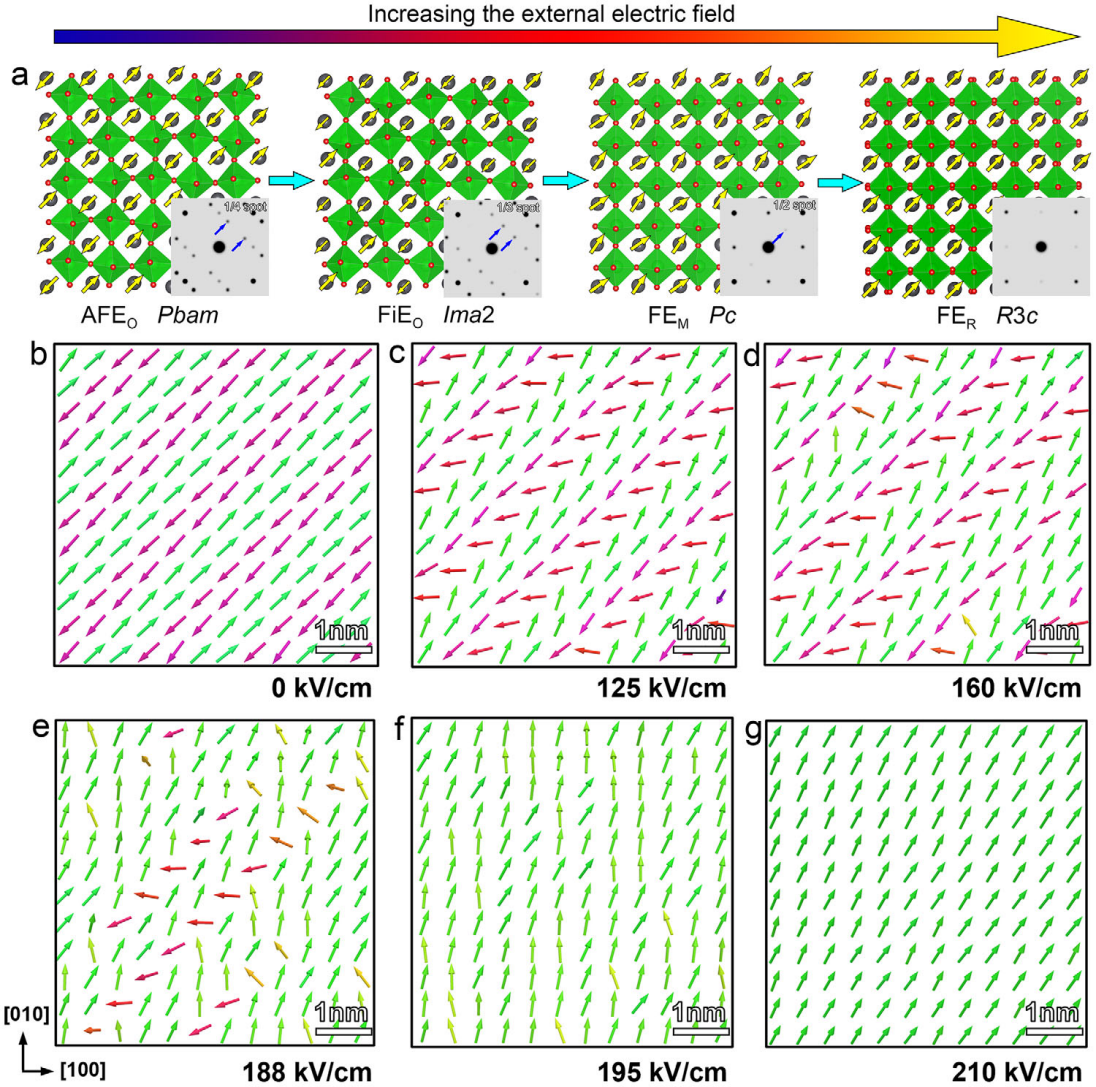
The phase transition pathway and molecular dynamics simulations based on the DeepMD model. (a) The schematic showing the electric‐field‐driven phase transition pathway. (b–g) Transient polarization evolution of PZO under progressively increasing electric field by molecular dynamics simulations. An external electric field is applied along the [001] crystallographic direction.

The complete electric‐field‐driven phase transition pathway in PZO films is schematically summarized in Figure 4a. At the initial state, the PZO exhibits a typical antiferroelectric phase (AFE_O_) with the space group *Pbam*. Upon increasing the electric field, the AFE_O_ phase undergoes a transformation into a ferrielectric phase (FiE_O_) with the space group *Ima*2. With further increase in the applied voltage, the FiE_O_ phase transitions into a monoclinic ferroelectric phase (FE_M_) with the space group *Pc*. Finally, under sufficiently high electric fields, the FE_M_ phase evolves into a rhombohedral ferroelectric (FE_R_) with the space group *R*3*c*. Therefore, the sequential phase transition pathway of antiferroelectric PZO under an external electric field proceeds from the AFE_O_ to FiE_O_, then to FE_M_, and ultimately to the FE_R_ phase. To validate the experimentally observed electric‐field‐driven phase transitions, molecular dynamics (MD) simulations were performed using an interatomic potential trained with the DeepMD framework [32], which constructs many‐body force fields from ab initio data via deep neural networks. Figure 4b–g displays the evolution of polarization configurations with the increasing external electric field. As illustrated in Figure 4b, in the absence of an applied electric field, the system adopts a ground‐state ↑↑↓↓ polarization configuration, characteristic of the AFE_O_ phase. As the external electric field is gradually applied along the [001] crystallographic direction, the antiparallel polarization vectors begin to rotate away from their initial [$\bar{1}\bar{1}0]$ orientation, reorienting nearly perpendicular to the applied field (Figure 4c). At a moderate field strength of 160 kV/cm (Figure 4d), partial reversal of polarization occurs, forming a local ↑↑↓ configuration. It indicates the emergence of a modulated intermediate phase. With further increase in field strength (Figure 4e), the polarization becomes progressively fragmented and spatially modulated. At approximately 195 kV/cm (Figure 4f), the polarization vectors exhibit a zig‐zag configuration, which is consistent with the monoclinic *Pc* phase. When the applied field reaches 210 kV/cm (Figure 4g), the system undergoes complete polarization reorientation along the [111] direction, stabilizing into a uniform rhombohedral *R*3*c* ferroelectric phase. At this point, all fractional diffraction spots vanish, confirming the disappearance of nanoscale polarization modulation and the establishment of long‐range rhombohedral order. These MD simulation results are in strong agreement with the experimental findings, thereby corroborating the proposed multi‐step electric‐field‐driven phase transition pathway from the antiferroelectric to ferroelectric state in PZO films. The result, achieved without any empirical parameters, demonstrates the applicability of machine‐learned potentials for modeling structural evolution in complex oxides.

The AFE‐to‐FE phase transition in antiferroelectric materials under an external electric field have significant impact on their energy storage performance. Although the phase evolution in antiferroelectric PZO under electron beam irradiation has been previously studied [18, 23], the intrinsic electric‐field‐induced phase transition behavior remains poorly understood. This limitation arises from the combined effects of charge accumulation, secondary electron emission, and introduction of oxygen vacancies during TEM observations [33], which obscures the pure electric‐field‐induced response. In this work, the electric‐field‐driven phase transitions in epitaxial PZO thin films were directly observed by in situ atomic‐resolution HAADF‐STEM imaging, which provides significant insights into the polarization reorientation under applied electric fields at the atomic scale. The phase transition pathway, from antiferroelectric (AFE_O_) to ferroelectric (FE_R_) phases, progresses through two distinct intermediate phases, which are ferrielectric (FiE_O_) and monoclinic ferroelectric (FE_M_) phases, respectively. A similar phase transition pathway has been reported in PZO/SrTiO_3_ films when the film thickness is reduced [21]. Previous studies have suggested that the FiE_O_ and FE_M_ interphases serve as crucial bridges between AFE_O_ and FE_R_ phases. These interphases enable a gradual reorientation of polarization from antiparallel to parallel configurations, thereby lowering the energy barrier and facilitating the AFE‐to‐FE phase transition along a low‐energy pathway [21]. Likewise, in our work, the emergence of FiE_O_ and FE_M_ interphases under an applied electric field can similarly reduce the energy barrier, making the AFE‐to‐FE transition easier to occur. Nevertheless, the fundamental driving forces underlying the two types of transitions differ. For the thickness‐dependent transition, the dominant factor is the surface effect as previously [21]. In contrast, an applied electric field generally reduces the electrostatic energy of the system [29, 34], which becomes the primary driving force for the electric‐field‐induced AFE‐to‐FE transition. Therefore, although both cases involve the formation of intermediate interphases, the phase transitions driven by the electric field and film thickness exhibit significant differences in their driving mechanisms. Moreover, the electric‐field‐induced AFE‐to‐FE phase transition behavior more closely reflects the real operating conditions of antiferroelectric materials, and elucidating this process is critical for optimizing their energy‐storage performance under practical working states.

A multi‐stage transition mechanism is energetically preferred over a direct one‐step AFE‐to‐FE transformation. Previous studies have reported that the FiE phase can coexist with the AFE ground state in both PZO single crystals and thin films [17, 18, 19], while the monoclinic ferroelectric phase has been observed in Nb‐doped Pb(Zr,Ti)O_3_ ceramics [31] and 15 nm thick PZO/SrTiO_3_ films [21]. These findings indicate that the two intermediate phases reside at relatively low energy states, as further evidenced by first‐principles density functional theory calculations [35]. Therefore, the sequential transition through these intermediate phases reduces the overall energy barrier, which leads to the multi‐stage process being more favorable.

Furthermore, this multi‐stage phase transition is accompanied by systematic evolution in diffraction patterns, polarization configurations, and lattice rotations, enabling a detailed understanding of the phase transition. Specifically, the superlattice reflections evolve sequentially from 1/4{110} to 1/3{110}, then to 1/2{110}, and ultimately to the disappearance of additional reflections. Correspondingly, the polarization modulation period gradually decreases stepwise from a quadruple‐modulated configuration to triple‐ and double‐modulated configuration, eventually culminating in a uniform polarization state. As schematized in Figure S6, this modulation collapse provides critical insight into the field‐induced phase transition. Fu et al. have reported that the modulation period increases with increasing Ti content and decreasing Sn content, which enhances the remanent polarization (P_r_) and decreases the forward switching field (E_AF_) due to the reduced interactions between adjacent dipoles with opposite directions [36]. In contrast, our observations reveal that the modulation period shortens as the electric field increases, which should correlate with a reduction in remanent polarization (P_r_) and an increase in the forward switching field (E_AF_). According to the energy storage density equation [37] $U_{e}=\int_{P_{r}}^{P_{m}}EdP$, this phase transition behavior can contribute to an enhancement in energy storage density. This indicates that the high energy storage performance of antiferroelectric materials is likely attributed to microscopically electric‐field‐driven multi‐step phase transitions. Therefore, our work established a clear relationship between microstructural evolution and functional properties through in situ electron microscopy, providing insights for further optimization of the energy storage performance of antiferroelectric materials.

## 3 Conclusion

In summary, we have demonstrated the atomic‐scale mechanism of the electric‐field‐driven AFE‐to‐FE phase transition in epitaxial PZO thin films by employing in situ aberration‐corrected STEM. The transition proceeds through a sequential evolution from an antiferroelectric state (AFE_O_) to an orthorhombic ferrielectric phase (FiE_O_), then to a monoclinic ferroelectric phase (FE_M_), and ultimately to a rhombohedral ferroelectric structure (FE_R_) with increasing electric field. This multi‐stage transition is unambiguously identified via fast Fourier transform (FFT) analysis, cation displacement mapping, and lattice rotations, and is further evidenced by machine learning molecular dynamics simulations. Importantly, this transition is accompanied by a gradual reduction in the polarization modulation period. Our findings may reveal a correlation between the microscopic phase transition pathway and the macroscopic energy storage behavior of PZO films. These insights not only advance the fundamental understanding of electric‐field‐induced phase transition dynamics in antiferroelectrics but also offer a clear strategy for optimizing the performance of high‐energy‐density capacitive devices.

## 4 Experimental Section

### 4.1 Film Deposition

The PZO thin films were deposited on (001)‐oriented SRO‐buffered KTO single‐crystal substrate via pulsed laser deposition (PLD). A sintered SRO target and a Pb‐enriched (3 mol%) PZO target were used to deposit SRO and PZO layers, respectively. Prior to deposition, the KTO substrates were heated to 800°C and held for 10 min to clean the substrate surface. Meanwhile, both targets were alternately pre‐sputtered for 5 min to eliminate surface contaminants. The SRO layer was deposited under the conditions with the substrate temperature of 680°C, an oxygen pressure of 7 Pa, laser energy of 350 mJ, and repetition rate of 4 Hz. For the PZO film deposition, the oxygen pressure was increased to 12 Pa while retaining the other parameters (the substrate temperature of 680°C, laser energy of 350 mJ, repetition rate of 4 Hz). After deposition, the samples were annealed at 680°C for 5 min under a high oxygen pressure of 3 × 10^4^ Pa. Finally, the films were cooled to room temperature at a controlled rate of 5°C/min.

### 4.2 TEM Samples Preparation

Cross‐sectional samples for STEM observations and in situ STEM experiments were prepared by standard mechanical and ion‐milling procedures, including gluing, mechanical grinding, dimpling, and Ar ion milling. Before the ion milling, the samples were thinned to 10 µm by using the Gatan Dimpling Grinder 656. The ion milling was performed with a Gatan Precision Ion Polishing System 695. The initial voltage of 4.5 kV and an angle of 7° were used. Then the voltage and angle gradually reduced in the milling process. The terminal voltage of 0.5 kV was applied to reduce the amorphous layer at the surface of the samples, which was generated by ion beam damage.

### 4.3 HAADF‐STEM Imaging

Atomic‐resolution HAADF‐STEM images were recorded by a double aberration‐corrected scanning transmission electron microscope (Spectra 300 X‐FEG microscope, ThermoFisher Scientific) at 300 kV. The STEM Drift Corrected Frame Integration (DCFI) technique was used to minimize the sample drift when acquiring atomic‐resolved HAADF‐STEM images [38]. Each atomic‐scale HAADF‐STEM image was reconstructed by adding up 20 sequential frames with a dwell time of 200 ns per frame using the Velox software (Thermo Fisher Scientific).

### 4.4 In Situ STEM Imaging

The in situ STEM experiments under external electric fields were conducted on an aberration‐corrected JEOL ARM 300F2 microscope equipped with a double‐tilt electric bias TEM holder (Zeptools Technology). The atomic‐scale HAADF‐STEM images were acquired in the microscope under STEM mode with the recorded parameters of 2048 × 2048 pixel for each frame and the dwell time of 2 µs for each pixel.

### 4.5 Atomic Columns Position Determination

The positions of atom columns in atomic‐scale HAADF‐STEM images were determined based on the 2D Gaussian fitting, which was carried out by using the Matlab software.

### 4.6 DP Model Training

The deep‐learning (DP) interatomic potential model for PZO was trained using the open‐source package DeePMD‐kit [32]. Initially, a seed dataset was generated based on five distinct polymorphs of DFT‐relaxed PZO: *Pm* $\bar{3}$ *m*, *Pbam*, *Ima*2, *R*3*c*, and *Pc*. Each structure was further perturbed by introducing random atomic displacements of up to ±0.2 Å, along with isotropic lattice scaling within a ±5% range. In addition, intermediate structures obtained by interpolating between the various polymorphic phases were included in the dataset. Additionally, short AIMD simulations were conducted using the Vienna ab‐initio simulation package (VASP) code [39] to enrich the configurational diversity of the dataset.

This seed dataset was then input into the DP‐GEN concurrent learning framework, which iteratively expanded the dataset via cycles of network training, DFT labeling, and configuration space exploration [40]. Within the DeepMD formalism, the total potential energy *E* of a configuration is expressed as a sum over atomic contributions *E_i_*, each derived from a local descriptor *D_i_* via an embedding neural network. The descriptor *D_i_* captures the local environment of atom *i* within a cutoff radius *R*_c_. To ensure smooth force predictions, a switching function was applied starting at 7.2 Å. The network architecture consists of an embedding network with layer widths of 25, 50, and 100 neurons, followed by a fitting network comprising three hidden layers, each with 240 neurons. Further details on the DeepMD network architecture can be found in the reference [41]. The final dataset supported DeepMD training in over 1 000 000 steps. The accuracy of energy and force predictions is demonstrated in Figure S7. The resulting potential accurately reproduces DFT‐level energies and structural transitions, offering a robust surrogate for large‐scale atomistic simulations.

### 4.7 MD Simulations

Classical molecular dynamics (MD) simulations were performed using LAMMPS in the NPT ensemble at 300 K with periodic boundary conditions, employing a 12 × 12 × 12 pseudocubic *Pbam* supercell (8640 atoms) [42]. A timestep of 1 fs was used, and system coordinates and velocities were recorded every 10 ps. External electric fields (ε) were applied using the force method, wherein an additional force $F_{i}$ is exerted on ion *i* as $F_{i}=Z_{i}^{*}\;\cdot E$, where $Z_{i}^{*}$ denotes the Born effective charge tensor obtained from DFT calculations. For simplicity and consistency, we employed the Born effective charges of the cubic phase in all simulations. These tensors were isotropic and diagonal with values: $Z_{\mathrm{Pb}}^{*}=3.90$, $Z_{\mathrm{Zr}}^{*}=5.85$, $Z_{O}^{*}=-3.25$.

## Author Contributions

X.L.M. and Y.L.Z. conceived the project on the architecture of quantum materials modulated by ferroelectric polarizations; X.L.M., Y.L.Z., and Y.P.F. designed the sample structure and subsequent experiments. Y.P.F. performed the statistical and in situ STEM observations. M.X.Z. and Y.J.W. performed a molecular dynamics simulation. R.J.J. performed the thin‐film growth and participated in the manuscript draft and revision. Y.L.T participated in the thin‐film growth and STEM observations. All authors participated in the discussion and interpretation of the data. Correspondence and requests for materials should be addressed to Y.L.Z. and X.L.M.

## Funding

Guangdong Basic and Applied Basic Research Foundation (No. 2023A1515011058), Guangdong Provincial Quantum Science Strategic Initiative (No. GDZX2302001, No. GDZX2402001), National Natural Science Foundation of China (No. 52501016, No. U24A2013, No. 52122101, No. 52471022), National Key Research and Development Program of China (2024YFA1408000), Liaoning Revitalization Talents Program (XLYC2203020, XLYC2403183), Science and Technology Major Project of Liaoning province (2024JH1/11700033), Youth Innovation Promotion Association CAS (2021187), Open research fund of Songshan Lake Materials Laboratory (2023SLABFK13), IMR Innovation Fund (2024‐ZD01).

## Conflicts of Interest

The authors declare no conflict of interest.

## Supporting information




**Supporting File**: advs73438‐sup‐0001‐SuppMat.docx.

## Data Availability

The data that support the findings of this study are available from the corresponding author upon reasonable request.
