# Supplementary material for: Direct Observation of Electric‐Field‐Driven Phase Transitions Associated with Energy Storage in Antiferroelectric Films
Source: Adv Sci (Weinh). 2025 Dec 21;13(13):e17897. doi: 10.1002/advs.202517897 (PMC12955928; doi:10.1002/advs.202517897)
Supplement: Supplementary file 1 — Supporting File: advs73438‐sup‐0001‐SuppMat.docx. [file ADVS-13-e17897-s001.docx]

Supporting Information

**Direct Observation of Electric-field-driven Phase Transitions Associated with Energy Storage in Antiferroelectric Films**

*Yan-Peng Feng, Mei-Xiong Zhu, Ru-Jian Jiang, Yu-Jia Wang, Yun-Long Tang,* *Yin-Lian Zhu***, and Xiu-Liang Ma**


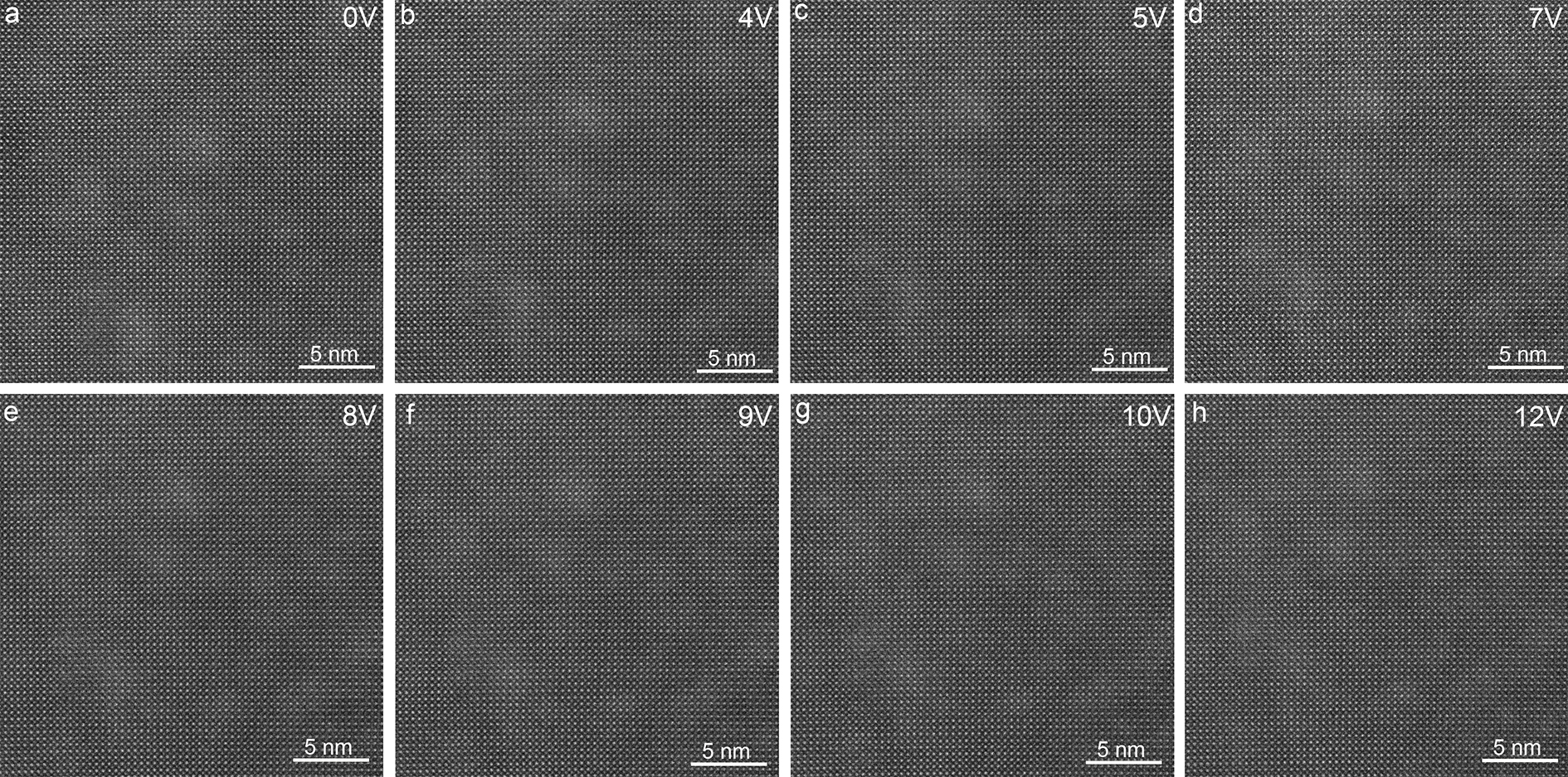


Figure S1. A series of original high-resolution HAADF-STEM images taken along [100] directions under different electric bias. (a) 0 V, (b) 4 V, (c) 5 V, (d) 7 V, (e) 8 V, (f) 9 V, (g) 10 V, (h) 12 V.


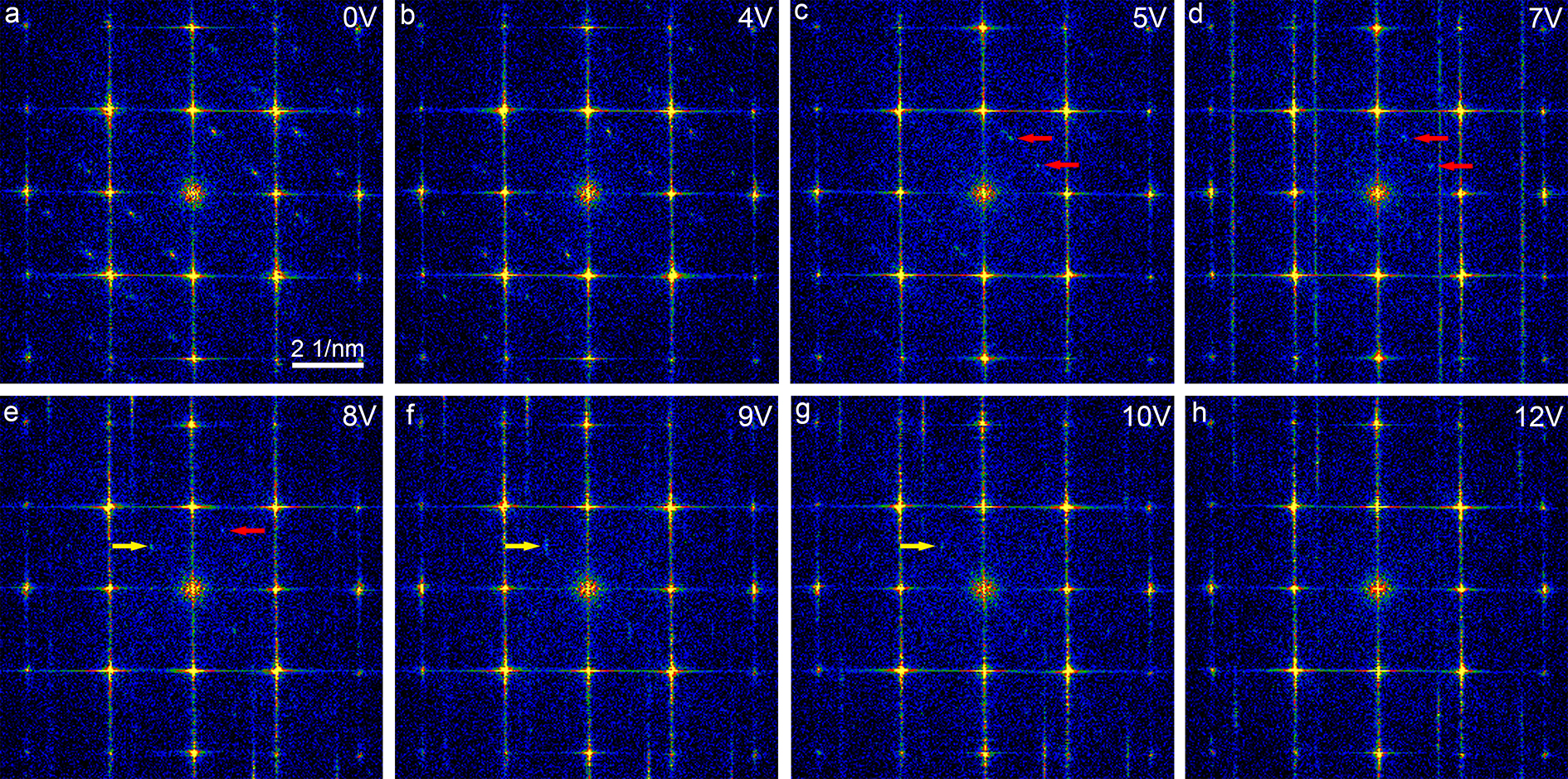


Figure S2. The FFT patterns corresponding to Fig. S1 reveal the phase transition in PZO films under external electric field. (a) The FFT pattern with additional 1/4{110} diffraction spots denotes the orthorhombic PZO phase under initial state. (b) The FFT pattern with 1/4{110} spots of PZO films showing no obvious change at 4 V. (c) The FFT pattern of PZO films at 5 V. The 1/3{110} diffraction spots (marked by red arrows) start to appear beside the 1/4{110} spots. (d) The FFT pattern of PZO films at 7 V. The 1/4{110} spots disappear and the 1/2{110} spots (marked by yellow arrows) start to appear at 7 V. (e-h) the FFT patterns of PZO films at 8 V (e), 9 V (f), 10 V (g), 12 V (h), respectively. The 1/3{110} spots disappear at 9 V. Finally, the 1/2{110} spots disappear at 12 V.


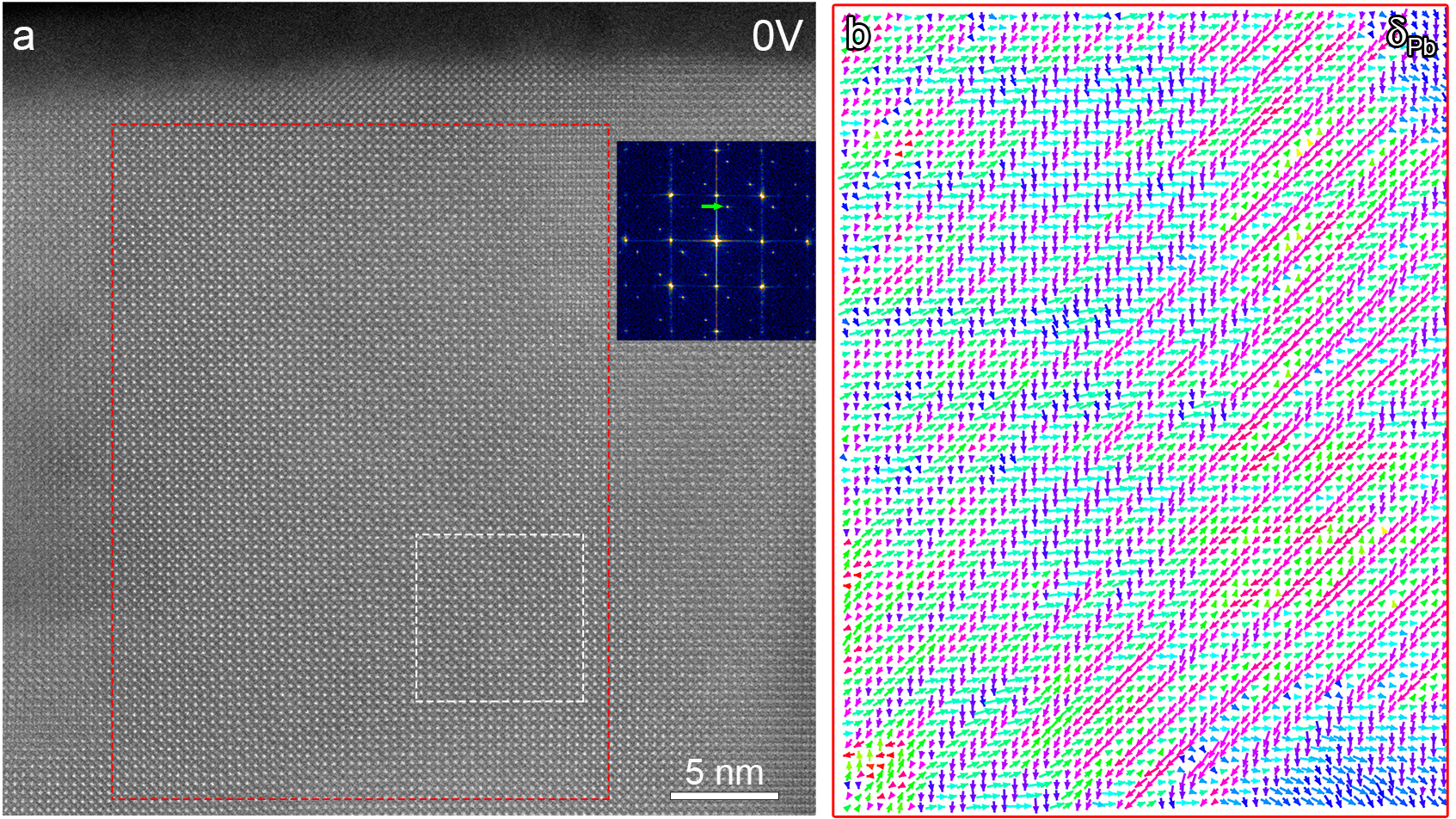


Figure S3. The atomic-resolved HAADF-STEM image and the polarization configuration at 0 V. (a) The atomic-resolved HAADF-STEM images of PZO films at 0 V. The inset is the FFT patterns corresponding to the regions of (a). The green arrow denotes an additional 1/4{110} reflection. (b) The Pb-displacement (**δ_Pb_**) mapping corresponding to the region labeled as dashed red rectangle box in (a).


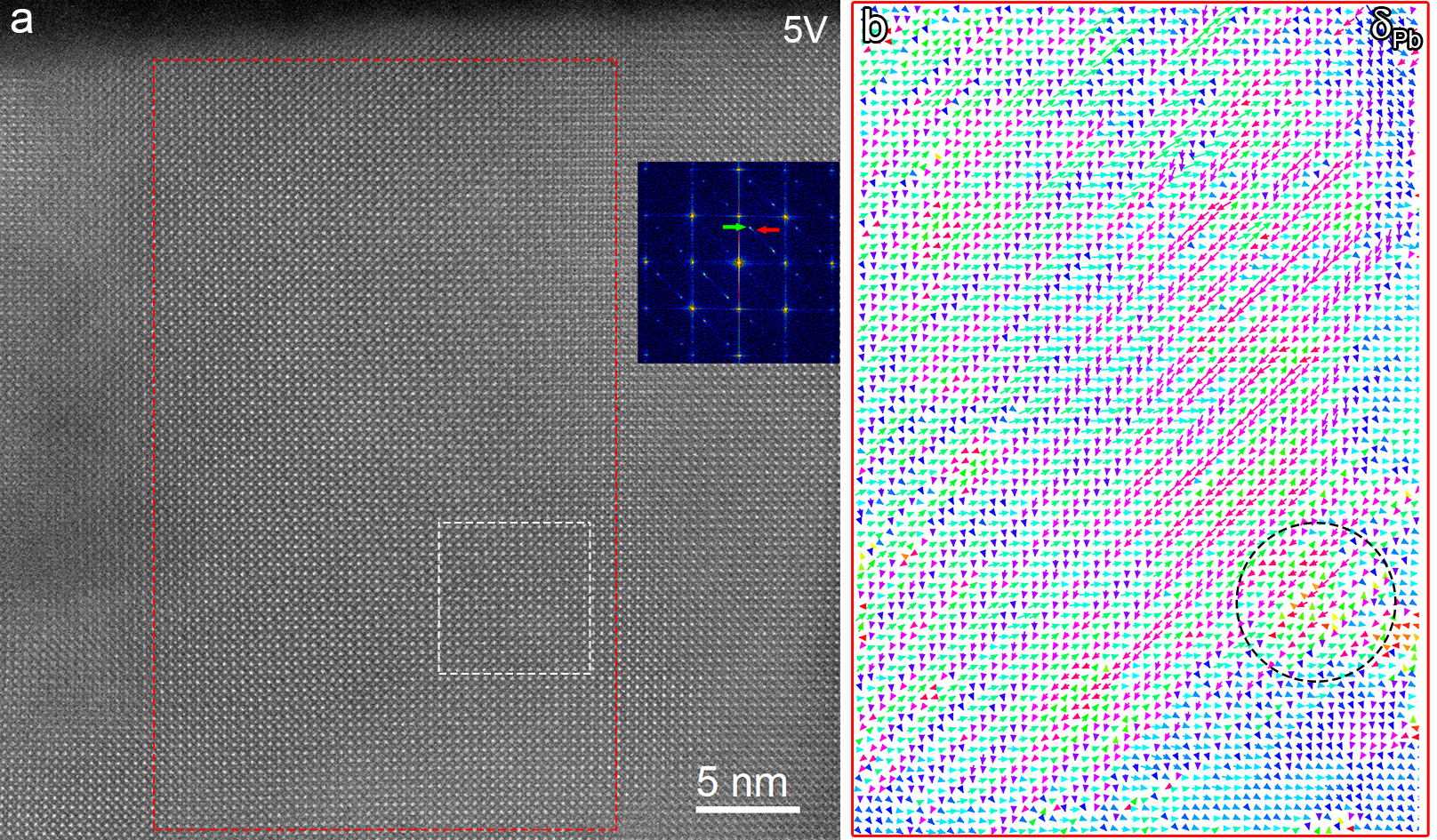


Figure S4. The atomic-resolved HAADF-STEM image and the polarization configuration at 5 V. (a) The atomic-resolved HAADF-STEM images of PZO films at 5 V. The inset is the FFT patterns corresponding to the regions of (a). The green and red arrows denote the additional 1/4{110} and 1/3{110} reflections, respectively. (b) The Pb-displacement (**δ_Pb_**) mapping corresponding to the region labeled as dashed red rectangle box in (a). The ferrielectric phase appears at the region marked by the dashed circle.


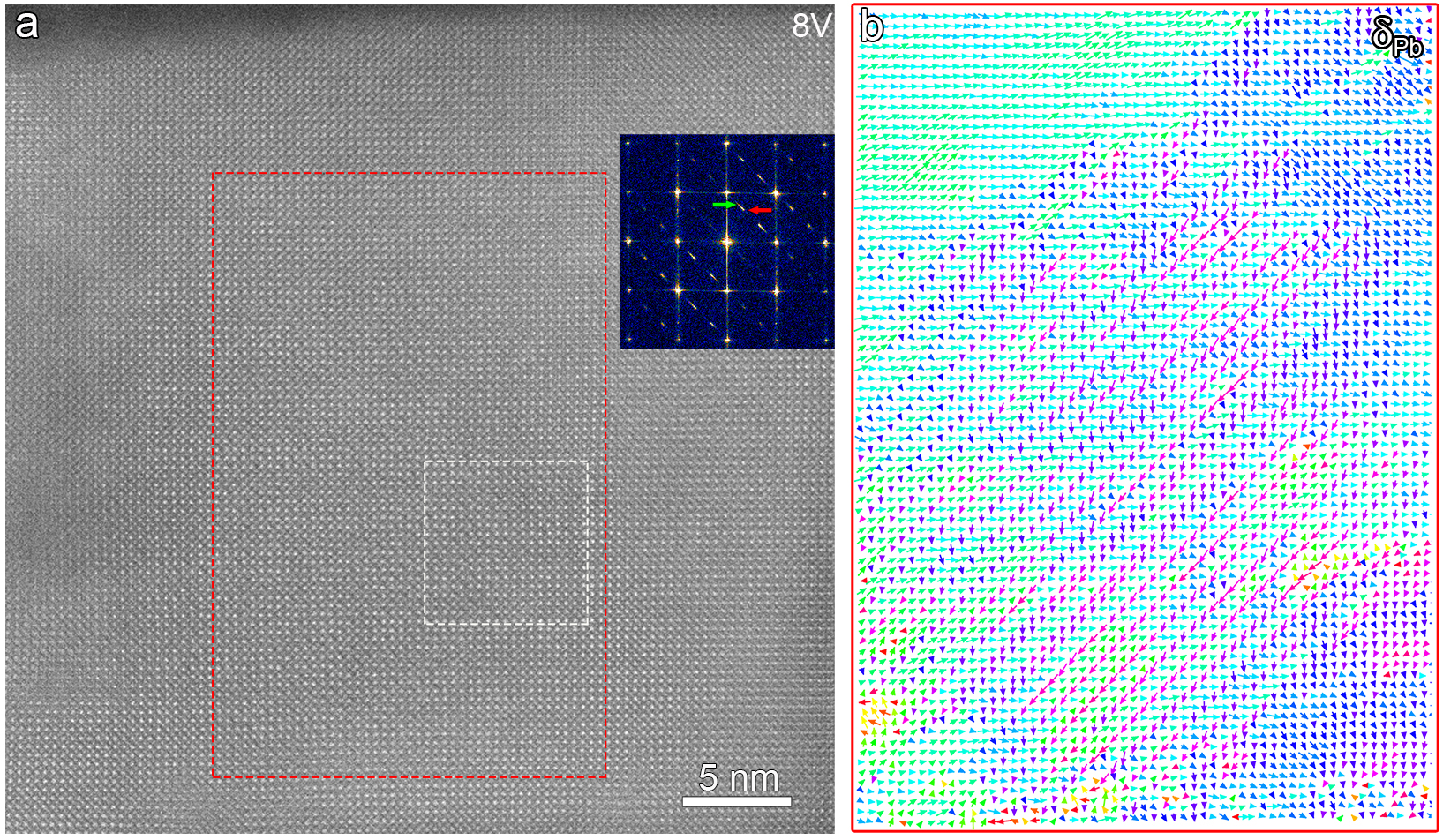


Figure S5. The atomic-resolved HAADF-STEM image and the polarization configuration at 8 V. (a) The atomic-resolved HAADF-STEM images of PZO films at 8 V. The inset is the FFT patterns corresponding to the regions of (a). The green and red arrows denote the additional 1/4{110} and 1/3{110} reflections, respectively. (b) The Pb-displacement (**δ_Pb_**) mapping corresponding to the region labeled as dashed red rectangle box in (a).


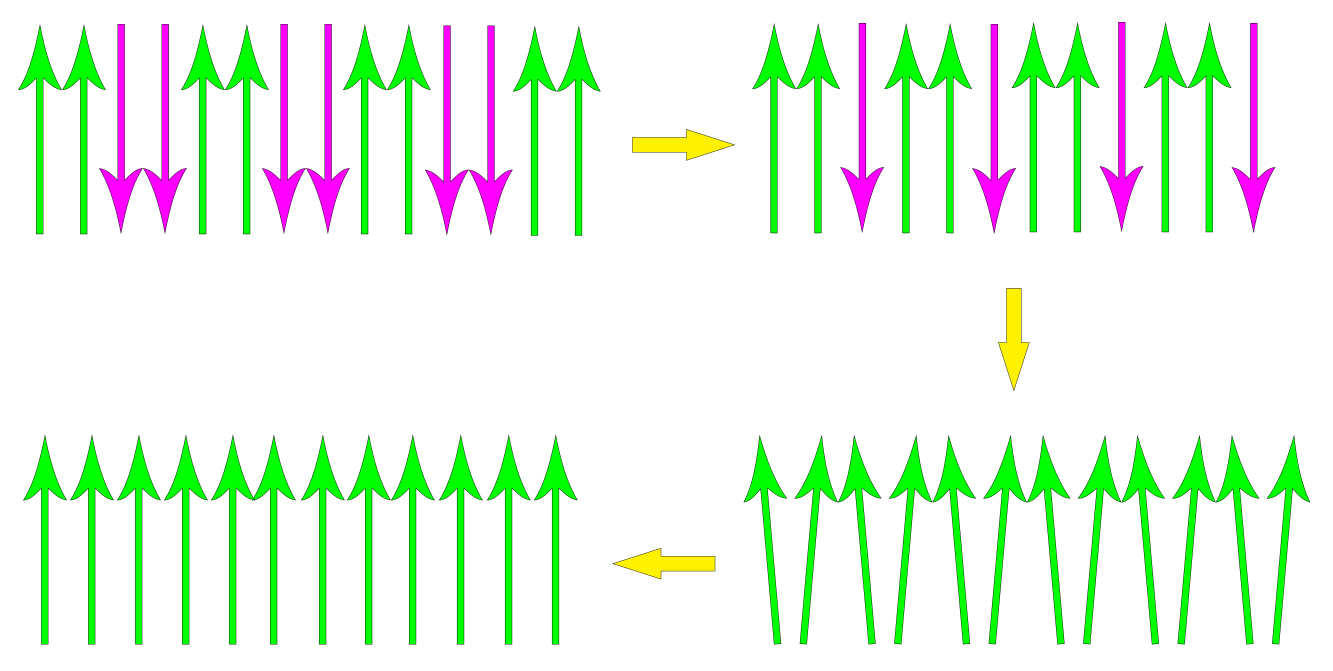


Figure S6. The evolution behavior of polarization modulation period in antiferroelectric PZO under external electric field.


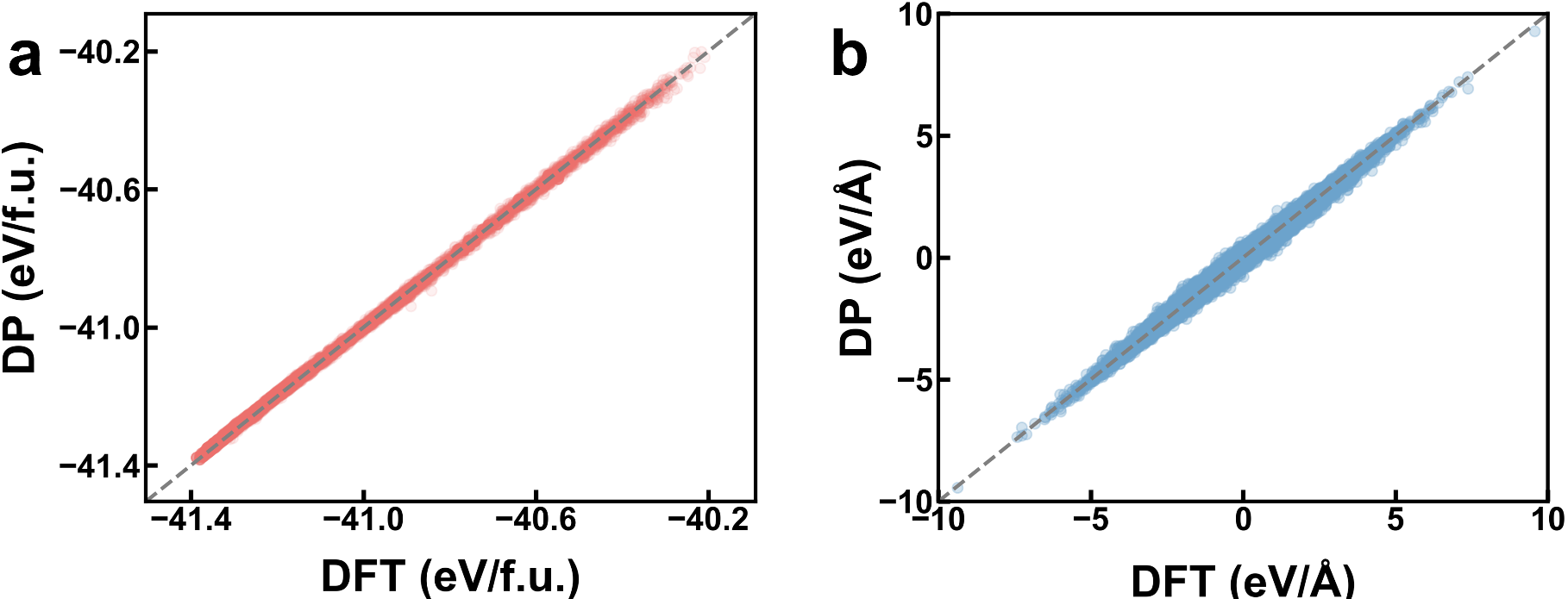


Figure S7. DP Model Validation on Final Training Database. Predicted (a) energies and (b) atomic forces from the DP model compared with DFT results for training configurations.
